# Supplementary material for: Empirical methods for controlling false positives and estimating confidence in ChIP-Seq peaks
Source: BMC Bioinformatics. 2008 Dec 5;9:523. doi: 10.1186/1471-2105-9-523 (PMC2628906; doi:10.1186/1471-2105-9-523)
Supplement: Additional File 1 — A variety of html documents from the USeq web site detailing the available applications, their best usage, output file type descriptions, command line menus, etc. [file 1471-2105-9-523-S1.zip › USeqUserGuides/JavaDoc.zip!/JavaDoc/bioroot/oligo/OligoModify.html]

OligoModify


|  |  |  |
| --- | --- | --- |
| **Overview** \| **Package** \| **Class** \| **Use** \| **Tree** \| **Deprecated** \| **Index** \| **Help** | |  |
| **PREV CLASS**   **NEXT CLASS** | **FRAMES**    **NO FRAMES**     **All Classes** |  |
| SUMMARY: NESTED \| FIELD \| CONSTR \| METHOD | DETAIL: FIELD \| CONSTR \| METHOD |  |


---


## bioroot.oligo Class OligoModify

```
java.lang.Object
  javax.servlet.GenericServlet
      javax.servlet.http.HttpServlet
          bioroot.oligo.OligoModify
```

**All Implemented Interfaces:**: java.io.Serializable, javax.servlet.Servlet, javax.servlet.ServletConfig

---

``` public class OligoModify extends javax.servlet.http.HttpServlet ```

Servlet to modify oligos using OligoBeans referenced from a Session object.

**Author:**
:   nix

**See Also:**: Serialized Form

---

**Constructor Summary**

`OligoModify()`


| **Method Summary** | |
| --- | --- |
| `static java.lang.String` | `deleteOligo(OligoBean oligoBean, UserBean userBean, DBUtil bioRoot)`             Attempts to delete an oligo, if sucessful, returns a "" String, otherwise an error message. |
| `void` | `doGet(javax.servlet.http.HttpServletRequest request, javax.servlet.http.HttpServletResponse response)` |
| `void` | `doPost(javax.servlet.http.HttpServletRequest request, javax.servlet.http.HttpServletResponse response)` |
| `static boolean` | `subtractAndSetOligoBeans(OligoBean[] oligoBeans, javax.servlet.http.HttpSession session)`             Subtracts a bean from the OligoBean[] and resets it in oligoBeansToEdit |
| `static java.lang.String` | `updateOligo(OligoBean oligoBean, UserBean userBean, javax.servlet.http.HttpServletRequest request, DBUtil bioRoot)`             Attempts to update an OligoBean in the database, only checks that the name is unique if it has changed. |

**Methods inherited from class javax.servlet.http.HttpServlet**

`service`

**Methods inherited from class javax.servlet.GenericServlet**

`destroy, getInitParameter, getInitParameterNames, getServletConfig, getServletContext, getServletInfo, getServletName, init, init, log, log`

**Methods inherited from class java.lang.Object**

`equals, getClass, hashCode, notify, notifyAll, toString, wait, wait, wait`

**Constructor Detail**

### OligoModify

```
public OligoModify()
```


**Method Detail**

### doGet

```
public void doGet(javax.servlet.http.HttpServletRequest request,
                  javax.servlet.http.HttpServletResponse response)
           throws javax.servlet.ServletException,
                  java.io.IOException
```

:   **Overrides:**: `doGet` in class `javax.servlet.http.HttpServlet`
:   **Throws:**: `javax.servlet.ServletException`: `java.io.IOException`

---


### doPost

```
public void doPost(javax.servlet.http.HttpServletRequest request,
                   javax.servlet.http.HttpServletResponse response)
            throws javax.servlet.ServletException,
                   java.io.IOException
```

:   **Overrides:**: `doPost` in class `javax.servlet.http.HttpServlet`
:   **Throws:**: `javax.servlet.ServletException`: `java.io.IOException`

---


### deleteOligo

```
public static java.lang.String deleteOligo(OligoBean oligoBean,
                                           UserBean userBean,
                                           DBUtil bioRoot)
```

:   Attempts to delete an oligo, if sucessful, returns a "" String, otherwise an error message.

---


### updateOligo

```
public static java.lang.String updateOligo(OligoBean oligoBean,
                                           UserBean userBean,
                                           javax.servlet.http.HttpServletRequest request,
                                           DBUtil bioRoot)
```

:   Attempts to update an OligoBean in the database, only checks that the name is unique if it has changed.
    If the update is sucessful, the message will be "", otherwise an error message will be returned.

---


### subtractAndSetOligoBeans

```
public static boolean subtractAndSetOligoBeans(OligoBean[] oligoBeans,
                                               javax.servlet.http.HttpSession session)
```

:   Subtracts a bean from the OligoBean[] and resets it in oligoBeansToEdit


---


|  |  |  |
| --- | --- | --- |
| **Overview** \| **Package** \| **Class** \| **Use** \| **Tree** \| **Deprecated** \| **Index** \| **Help** | |  |
| **PREV CLASS**   **NEXT CLASS** | **FRAMES**    **NO FRAMES**     **All Classes** |  |
| SUMMARY: NESTED \| FIELD \| CONSTR \| METHOD | DETAIL: FIELD \| CONSTR \| METHOD |  |


---
